# Early cell-autonomous accumulation of neutral lipids during infection promotes mycobacterial growth

**DOI:** 10.1371/journal.pone.0232251

**Published:** 2020-05-14

**Authors:** Colleen M. McClean, David M. Tobin

**Affiliations:** 1 Department of Molecular Genetics and Microbiology, Duke University School of Medicine, Durham, North Carolina, United States of America; 2 Department of Immunology, Duke University School of Medicine, Durham, North Carolina, United States of America; 3 Medical Scientist Training Program, Duke University School of Medicine, Durham, North Carolina, United States of America; Institut de Pharmacologie et de Biologie Structurale, FRANCE

## Abstract

Lipids represent an important source of nutrition for infecting mycobacteria, accumulating within the necrotic core of granulomas and present in foamy macrophages associated with mycobacterial infection. In order to better understand the timing, process and importance of lipid accumulation, we developed methods for direct *in vivo* visualization and quantification of this process using the zebrafish-*M*. *marinum* larval model of infection. We find that neutral lipids accumulate cell-autonomously in mycobacterium-infected macrophages *in vivo* during early infection, with detectable levels of accumulation by two days post-infection. Treatment with ezetimibe, an FDA-approved drug, resulted in decreased levels of free cholesterol and neutral lipids, and a reduction of bacterial growth *in vivo*. The effect of ezetimibe in reducing bacterial growth was dependent on the *mce4* operon, a key bacterial determinant of lipid utilization. Thus, *in vivo*, lipid accumulation can occur cell-autonomously at early timepoints of mycobacterial infection, and limitation of this process results in decreased bacterial burden.

## Introduction

During the course of *Mycobacterium tuberculosis* (*Mtb*) infection, bacilli are taken up by host macrophages. Persistent infection is established, in part, by bacterial manipulation of these macrophages in order to reside and replicate within them [1]. Infected and uninfected macrophages form characteristic aggregated structures called granulomas [2] that, when mature, consist of characteristic layers of epithelialized macrophages organized around a lipid-laden necrotic core [3]. While initially thought to serve as exclusively host protective structures, granulomas not only contain infection, but also represent a protected niche in which bacteria may replicate, shielded in part from the adaptive immune system [2–4]. The granuloma spatially restricts infection but also provides a site for infection of previously uninfected macrophages, which may then disseminate to new sites; in addition, the granuloma represents a nutritionally unique space [2, 5, 6]. Extensive research has established the importance of lipids and cholesterol as nutritional sources during infection [7]. *Mtb* bacilli have been show to accumulate and utilize triglycerides acquired from host macrophages [8] and it has also been shown that host cholesterol is required in order to sustain persistent mycobacterial infection [9, 10].

In addition, lipid-laden or foamy macrophages (FM) have been described as a hallmark of the tuberculous granuloma since the first pathological descriptions of this canonical structure were made more than 100 years ago [11]. Recent work has demonstrated that particularly pathogenic and not saprophytic mycobacteria induce the formation of FMs [12] suggesting that these cells develop as a consequence of infection and are associated with pathogen virulence. These cells accumulate intracellular lipid droplets (LDs) composed primarily of neutral lipids, principally cholesteryl esters (CE) and triglycerides (TAG) within macrophages [13, 14]. It has been hypothesized that foamy macrophages develop from the lipid-rich necrotic core of mature granulomas as these cell types have largely been observed in mature granulomas and proximal to the necrotic core [11].

In order to examine the dynamics of lipid accumulation during mycobacterial infection in vivo we developed a Fluorescence-Activated Cell Sorting (FACS) approach and employed high-resolution microscopy using the zebrafish-*M*. *marinum* model of infection. This model has been previously used to describe granuloma dynamics and formation [3, 15, 16], macrophage biology, as well as lipid metabolism and storage, displaying signaling pathways conserved with those of humans [17–19]. In addition, mycobacterial pathogenesis in zebrafish closely resembles many aspects of human tuberculosis including macrophage infection, aggregation, and even foam cell formation [3, 20–22].

In the present study, we demonstrate that lipids accumulate cell-autonomously in infected macrophages during mycobacterial infections *in vivo* and that this accumulation begins to take place at relatively early timepoints. We treated zebrafish with ezetimibe in order to reduce available free cholesterol and neutral lipids during early infection and showed that this treatment results in an *in vivo* bacterial growth defect compared to untreated animals. Using *M*. *marinum* strains defective in lipid utilization, we show that the *in vivo* growth restriction of bacteria by ezetimibe depends on a functional *mce4* operon. Taken together these data suggest that lipid accumulation specifically in infected macrophages during early mycobacterial infection can confer a direct benefit to growing bacteria. Manipulation of the ability of mycobacteria to accumulate lipids during early infection may represent a relevant mechanism to control the progression of mycobacterial infections.

## Results

### Neutral lipids accumulate cell-autonomously in *M*. *Marinum*-infected macrophages

**Fig 1:**
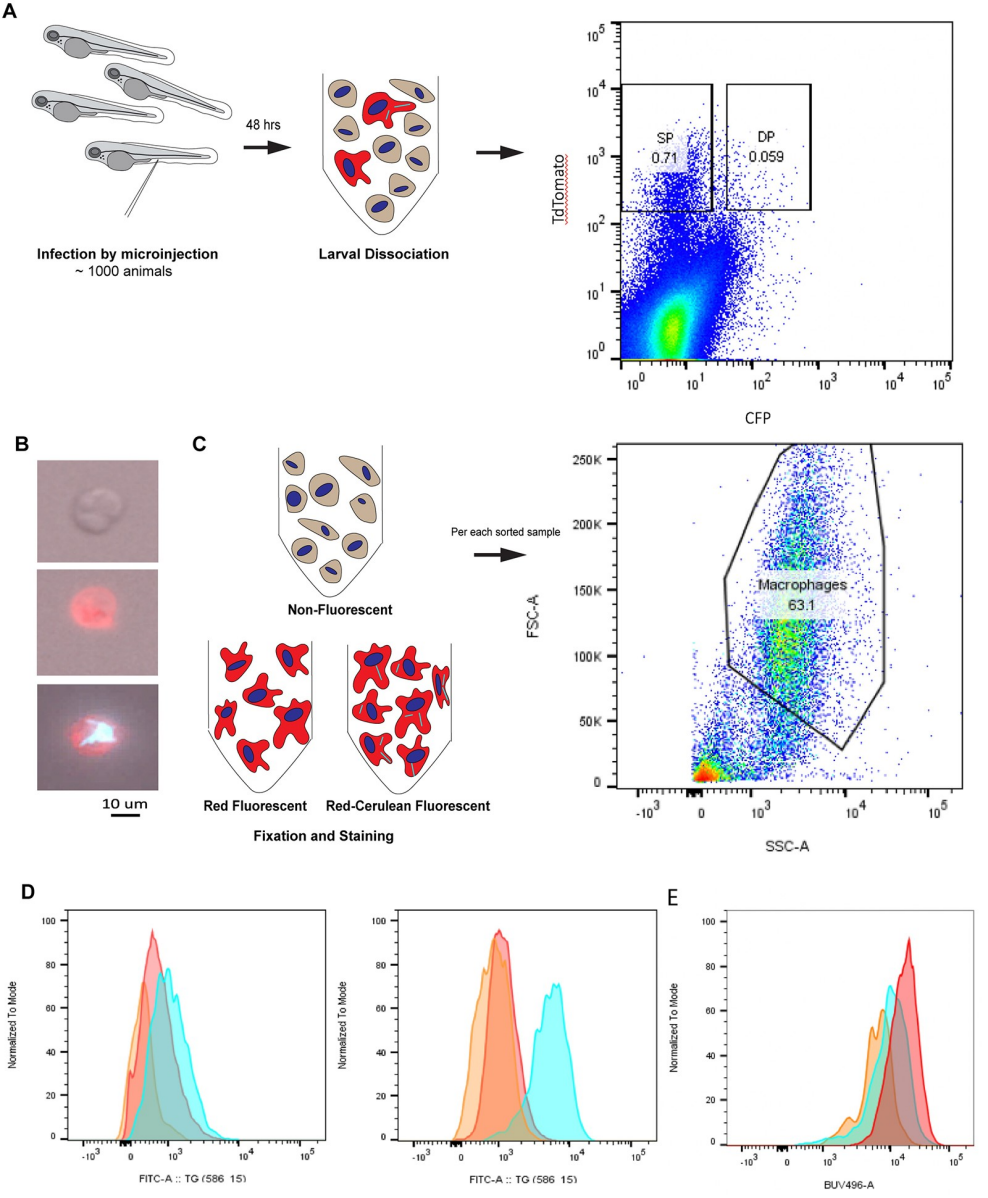
Neutral lipids accumulate cell-autonomously in *M*. *marinum* infected macrophages by 48 hours post-infection. **a**. Schematic diagram representing infection of 2 dpf larvae through the caudal vein, followed by cell dissociation after 48 hrs and sorting of single positive (SP, red) and double positive (DP, red-cerulean) macrophages from non-fluorescent cells. **b**. Epifluorescent images of representative sorted cells; non-fluorescent, red fluorescent, and red-cerulean fluorescent, respectively, from top to bottom. **c**. Schematic diagram representing fixation and staining of sorted cell types followed by analysis of intact cells gated on the basis of size exclusion. **d**. Measurement of Nile red fluorescence in sorted cell populations by analysis following excitation at 488 nm and measurement of emission via a 586/15 bandpass filter of non-fluorescent (orange), uninfected (red) and infected (cerulean) macrophages. Increases from 2.1- to 12.4- fold were observed in the geometric mean fluorescence intensity of Nile red staining between uninfected and infected macrophages. Histograms shown are the minimum and maximum fold changes observed among four independent replicates. Two additional replicates are shown in S1 Fig. **e**. Measurement of Filipin III fluorescence in sorted cell populations by analysis following excitation at 360 nm and measurement of emission via a 515/30 bandpass filter of non-fluorescent (orange), uninfected (red) and infected (cerulean) macrophages. No reproducible increase in fluorescence of infected macrophages compared to uninfected macrophages was observed. The histogram shown is representative of four independent replicates. Each independent experiment consisted of approximately 1000 infected larvae.

In order to assess the presence and timing of cholesterol and neutral lipid accumulation within macrophages during *M*. *marinum* infection, we examined the accumulation of both free cholesterol and neutral lipids (which include both cholesterol esters and triacylglycerol) in infected and uninfected macrophages *in vivo*. We infected zebrafish embryos at two days post-fertilization (dpf) and allowed infection to proceed. We used the *Tg*(*mfap4*:*TdTomato-CAAX)*^*xt6*^ transgenic line in which membrane-localized TdTomato expression is driven by the macrophage specific *mfap4* promoter [21]. We infected *Tg*(*mfap4*:*TdTomato-CAAX)*^*xt6*^ larvae with cerulean-expressing *M*. *marinum*, allowed the infection to develop over 48 hours, euthanized the animals, and then subjected the animals to cellular dissociation. Using FACS, we then sorted dissociated cells for the presence of the two fluorescent signals in order to obtain distinct populations of uninfected and infected macrophages separated from all other cell types (Fig 1A). In order to validate our sorting method, we spotted specific sorted cells and imaged them via epifluorescence microscopy (Fig 1B). We found that we obtained separate populations of infected macrophages, uninfected macrophages, and other cell types (Fig 1B).

We next used the fluorescent dyes Filipin and Nile red in order to quantify relative accumulation of free cholesterol and neutral lipids, respectively, in the sorted populations. Filipin has been previously validated as a probe to identify unesterified or free cholesterol during cellular sorting [23] in cultured macrophages whereas Nile red has been used to visualize lipid containing structures in live zebrafish [24] and is validated as a probe for neutral lipids generally [25]. Neutral lipids stained by Nile red include both triacylglycerols/triglycerides (TAG) and steryl esters/cholesterol esters (CE) [26, 27], whereas filipin stains free cholesterol specifically [28].

We fixed and stained sorted cell populations and then analyzed these previously sorted cell populations for relative filipin and Nile red signal. We observed a cell-autonomous increase in Nile red signal in infected macrophages compared with uninfected macrophages from infected larvae as well as sorted macrophages from control animals. In four independent experiments, consisting of sorts from approximately 1000 infected larvae each, the increase in Nile red signal in infected macrophages compared to uninfected macrophages from the same animals ranged from 2 to 12.4 fold (Fig 1D), depending on the experiment, with intermediate fold-change increases of 4 (S1A Fig) and 3.5 (S1B Fig). We observed no significant reproducible change in filipin signal between infected and uninfected macrophages (Fig 1E) in these analyses, consistent with most changes in cellular cholesterol content relating to cholesterol esters. These results show that there is a cell-autonomous increase in neutral lipid accumulation in infected macrophages indicating an accumulation in esterified cholesterols and/or TAGs, but no detectable accumulation of free cholesterol. This increase begins during the early stages of mycobacterial infection, with differences observable as early as 48 hours after the introduction of bacteria.

### Lipid accumulation occurs during *in vivo* infections

**Fig 2:**
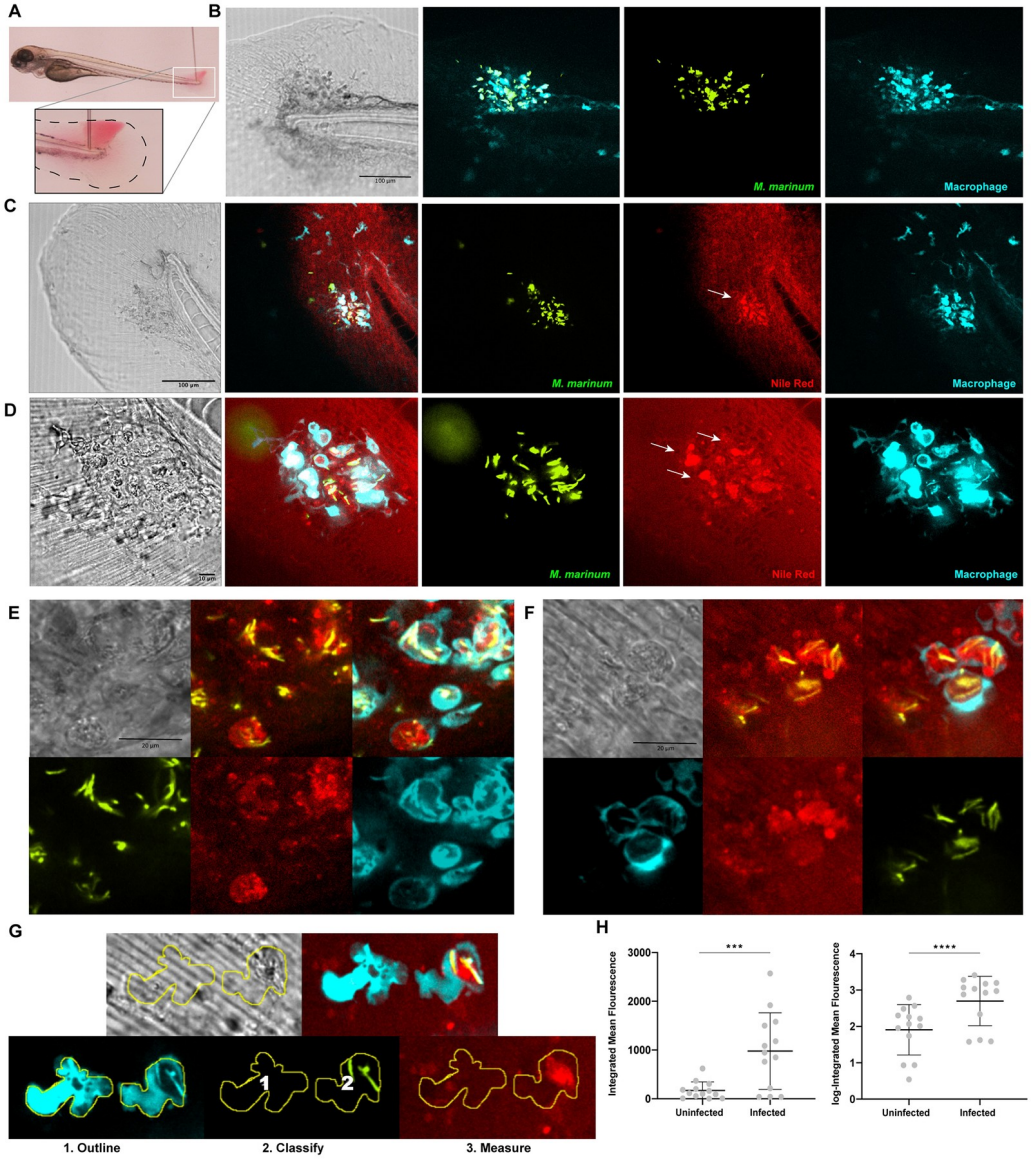
Neutral lipid accumulation is observed during *in vivo* infections using vital staining. **a**. Larvae were injected at 3 dpf into the tail fin. A bacterial suspension was deposited between epithelial layers of the tail fin as shown. **b**. Confocal imaging of the tail fin of infected *Tg(mfap4*:*p2A-Turquoise2)* larvae at 2 days post infection (dpi) with *M*. *marinum* (green) showing macrophage (turquoise) recruitment to site of infection. Bar = 100 μm. **c**. Confocal imaging of *Tg(mfap4*:*p2A-Turquoise2)* larvae 2 dpi with *M*. *marinum* (green) and following staining with Nile red. Arrows indicate location of neutral lipid accumulation within infected macrophages. Bar = 100 μm. **d**. Confocal imaging of *Tg(mfap4*:*p2A-Turquoise2)* larvae 2 dpi with *M*. *marinum* (green) and following staining with Nile red. Arrows indicate location of neutral lipid accumulation within infected macrophages. 60x objective, bar = 10 μm. **e**. Confocal imaging of a representative *Tg(mfap4*:*p2A-Turquoise2)* larva 2 dpi with *M*. *marinum* (green) and following staining with Nile red showing detail of Nile red staining pattern. 60x objective, bar = 20 μm. **f**. Confocal imaging of a representative *Tg(mfap4*:*p2A-Turquoise2)* larva 2 dpi with *M*. *marinum* (green) followed by staining with Nile red showing detail of Nile red staining pattern. 60x objective, scale bar = 20 μm. g. Representative images of uninfected and infected macrophages depicting the method of quantification of Nile red fluorescence intensity by first outlining all macrophages, then classifying uninfected (1) vs. infected (2) macrophages, and then measuring the integrated mean Nile red fluorescence of uninfected vs. infected macrophages. **h**. Integrated mean fluorescence and log-Integrated mean fluorescence of Nile red in uninfected vs. infected macrophages. Each data point represents the mean value of all macrophages within each class for a given animal. Data were collected from 13 individual animals. ***p = 0.0002 (Wilcoxon matched-paired sign rank test), ****p<0.0001 (paired t-test).

Given the observation that lipid accumulation begins early during mycobacterial infection, we next wanted to confirm that the observed accumulation occurs *in vivo*. We hypothesized that we could visualize neutral lipid accumulation intravitally in subcellular structures during early larval zebrafish infections by establishing infections in a particularly accessible region of infection. We adapted a protocol to infect 3 day post-fertilization (dpf) larval zebrafish in the tail fin as previously described [29] and then stained these larvae with Nile red in order to identify areas of neutral lipid accumulation (Fig 2A). By infecting into the larval tail fin, a thin two layer epithelial structure to which macrophages can be recruited during infection, we were able to directly visualize infected macrophages, including subcellular structures, with more accessibility than in thicker parts of the animal. Other fluorescent dyes commonly used as vital lipid markers including the BODIPY 505/515, BODIPY 500/510 C, and BODIPY 493/503 were tested and found to penetrate this tissue region poorly and therefore not useful at this timepoint and region.

We used the *Tg*(*mfap4*:*Turquoise2)*^*xt27*^ transgenic line in which macrophages are labeled turquoise and infected with wasabi-expressing *M*. *marinum*. We were able to infect the tail fins of larval zebrafish and observe recruitment of macrophages into the fin establishing a focus of infection accessible for vital staining and imaging (Fig 2B). We allowed the infection to develop over 48 hours and then stained live animals with Nile red. We then observed the relative abundance of Nile red in infected cells where *M*. *marinum* bacilli were present and in macrophages where bacilli were not present (Fig 2C and 2D). We observed an increase in Nile red staining in areas were *M*. *marinum* bacilli were present within macrophages (Fig 2E and 2F, S2A and S2B Fig). Of note, we observed no similar increase of Nile red staining in tail fins that were mock infected (S3A Fig), although we did observe some recruitment of macrophages to the location of mock infection or wounding.

In order to provide a quantitative measure of the difference in neutral lipid accumulation during early *M*. *marinum* infection *in vivo* we first identified and outlined all macrophages visualized during tail fin infections using image analysis software. We then classified these macrophages as either infected or uninfected on the basis of visualization of internalized mycobacterial bacilli (Fig 2G). We measured the relative Nile red signal in each macrophage and then generated the mean integrated Nile red fluorescence intensity of infected versus uninfected macrophages plotting these values for each infected animal. Notably we did not detect any significant Nile red staining of the bacteria themselves. Comparing the integrated mean fluorescence and log-integrated mean fluorescence of Nile red between uninfected and infected macrophages revealed a significantly elevated Nile red signal in infected macrophages as compared to uninfected macrophages for the same animals (Fig 2H). These results suggest that neutral lipids accumulate specifically within mycobacterium-infected macrophages, beginning during the early stages of infection. The ability to observe this accumulation live during *M*. *marinum* infections without manipulation of the environment of infection implicates early lipid accumulation as a feature of the natural pathogenesis of mycobacterial infection.

### Pharmacological reduction of environmentally available lipids is associated with reduced infection burden

**Fig 3:**
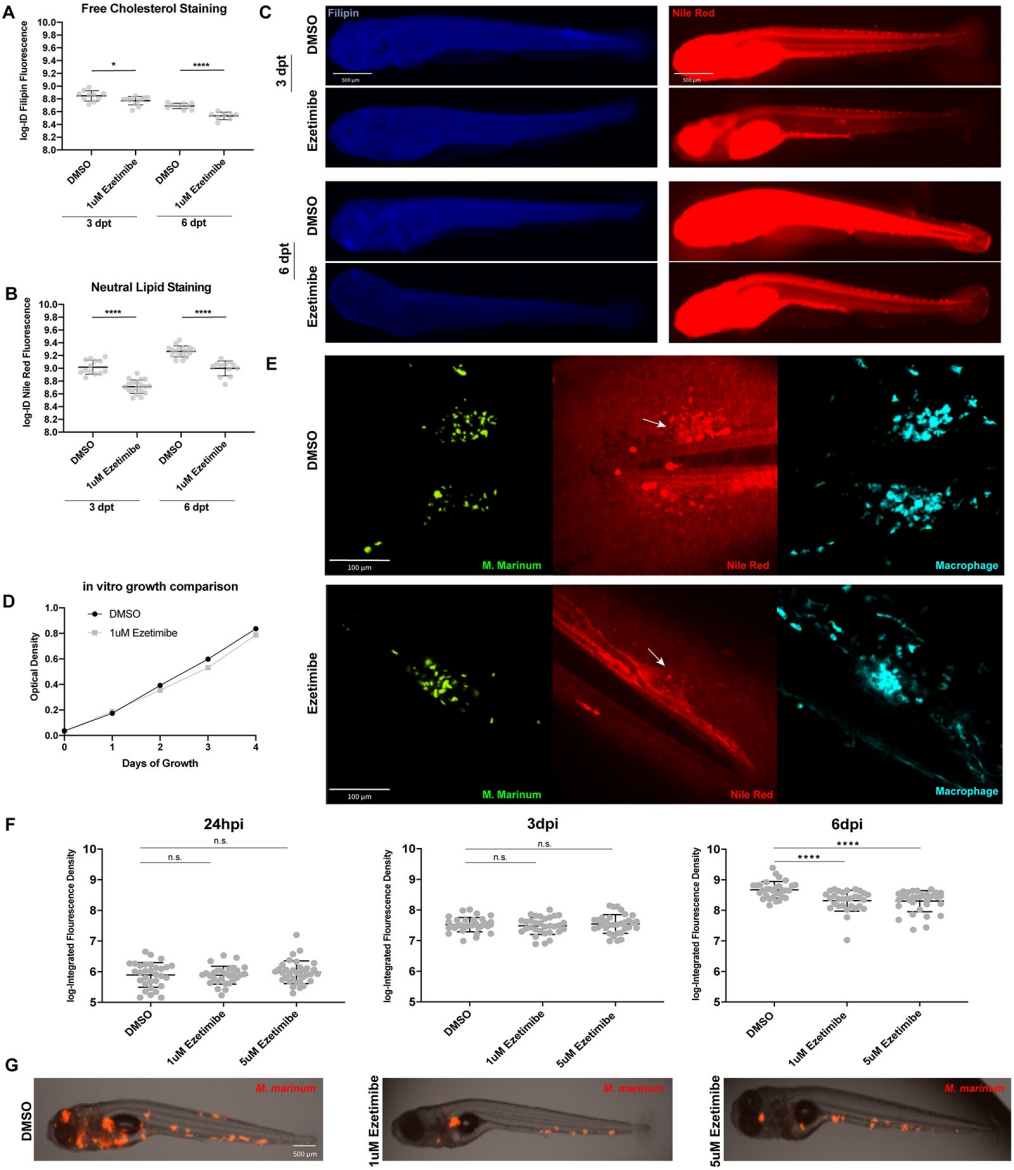
Ezetimibe treatment results in reduced neutral lipid accumulation and reduced infection burden. **a**. Comparison of integrated density of filipin fluorescence in the tail region of animals treated with vehicle alone (0.5% DMSO) or 1 μM ezetimibe. Data were collected at 3 and 6 days post-treatment (dpt). Student’s t-test *p = 0.018, ****p<0.0001 **b**. Comparison of integrated density of Nile red fluorescence in the tail region of animals treated with vehicle alone (0.5% DMSO) or 1 μM ezetimibe. Data were collected at 3 and 6 dpt. Student’s t-test ****p<0.0001 **c**. Animals representing the median values from comparison groups in (a) and (b). Bar = 500μm. **d**. Four-day time course of bacterial growth *in vitro*. Culture was maintained in either vehicle alone (0.5% DMSO) or 1 μM ezetimibe. No significant differences in bacterial growth were observed, Pearson R-squared = 0.99. **e**. Representative images of larval tail fins 2dpi in the absence or presence of ezetimibe treatment. Arrows indicate location of infection foci with (DMSO) or without (ezetimibe) neutral lipid accumulation. Bar = 100μm **f**. Comparison of infection burden at 24 hpi, 3 dpi, and 6 dpi in animals treated with vehicle alone (DMSO), 1 μM and 5 μM ezetimibe. No statistically significant differences were observed between groups at 24 hpi and 3dpi. One- way ANOVA with Tukey’s multiple comparisons ****p<0.0001. Representative of four independent experiments. **g**. Animals representing the median values from 6 dpi comparison groups in (f).

Given our observation that lipid accumulation occurs cell-autonomously within infected macrophages during early infection, we asked whether reduction of available lipids would 1) result in a reduction or loss of this observed lipid accumulation and 2) whether this loss of accumulation would result in a reduction in mycobacterial growth. In order to test this hypothesis we used the drug ezetimibe, an FDA-approved medication used to treat hypercholesterolemia in human patients that functions by blocking the uptake of cholesterol and other lipids from the digestive tract [30]. Ezetimibe has also been previously shown to reduce free cholesterol levels in zebrafish [31–33] but had not been tested as to its potential to reduce overall neutral lipid bioavailability. We first demonstrated that treatment of larval zebrafish with 1 μM ezetimibe reduced both available free cholesterol (Fig 3A and 3C) and to an even greater extent available neutral lipids (Fig 3B and 3C), as measured by the integrated fluorescence density of filipin and Nile red staining of whole treated larvae respectively at both 3 and 6 days post-treatment.

We then sought to determine whether treatment of larval zebrafish with ezetimibe and the corollary decrease in available lipids would result in a decreased accumulation of neutral lipids at infection foci and/or an overall decrease in infection burden using the tail fin model of infection. To ensure that any effect on infection was host-directed, we first tested the effect of ezetimibe on mycobacterial growth *in vitro* (Fig 3D) and observed no difference in the *in vitro* growth rate of wildtype *M*. *marinum* when exposed to vehicle alone (0.5% DMSO) or 1 μM ezetimibe over a 4-day time-course.

We infected 3 dpf *Tg*(*mfap4*:*Turquoise2)*^*xt27*^ transgenic larval zebrafish with wasabi-expressing *M*. *marinum* in the tail fin and immediately exposed infected larvae to either 0.5% DMSO or 1 μM ezetimibe. We then examined the larvae for accumulation of neutral lipids at the focus of infection and observed a loss of previously described neutral lipid accumulation proximal to bacilli in the tail fin model of infection (Fig 3E). We quantitated the Nile red signal at the foci of infection both in animals treated with 1 μM ezetimibe in 0.5% DMSO or vehicle alone. We found there to be a significant reduction in Nile red signal at the infection foci in ezetimibe-treated animals compared to animals without treatment (S4 Fig, p = 0.0076). These results suggest that the reduction in available neutral lipids observed during whole animal treatment with ezetimibe (Fig 3B and 3C) correlates with reduced accumulation of neutral lipids within mycobacterium-infected macrophages at the focus of infection.

We then sought to assess the effect of ezetimibe treatment on *M*. *marinum* infection burden within the zebrafish model of infection. We infected 2 dpf wild-type larvae with TdTomato-expressing *M*. *marinum* and then exposed infected larvae to either 0.5% DMSO, 1 μM ezetimibe, or 5 μM ezetimibe. We measured initial infection burden in the three assessment groups at 24 hours post infection (hpi) and observed no difference in the initial infection in each group. We followed infection to 6 days post infection (dpi) measuring infection burden at 3 dpi and 6 dpi timepoints. No difference in infection burden was observed by 3 dpi, but a significant reduction in infection burden was observed in both the 1 μM and 5 μM ezetimibe treatment groups by 6 dpi (Fig 3F). This result was also independently replicated by another lab member (E. Hughes). Visualization of infection by epifluorescent microscopy at the 6 dpi timepoint revealed decreased burden, and a reduction in the number of infection foci and distribution of infection in the ezetimibe treated animals (Fig 3G).

### Mutations in *M*. *marinum mce4* operon genes eliminate the effect of pharmacological reduction of neutral lipids.

The *mce4* operon is composed of 10 related genes controlled by a single promoter and has been identified as a source of membrane-associated and exported mycobacterial proteins [34]. This operon was previously described to be required for survival during stages of mycobacterial infection after the onset of an adaptive immune response [35, 36] and has been shown to encode the major cholesterol import system in mycobacteria [9], which allows mycobacteria to utilize cholesterol as a carbon and energy source [35].

**Fig 4:**
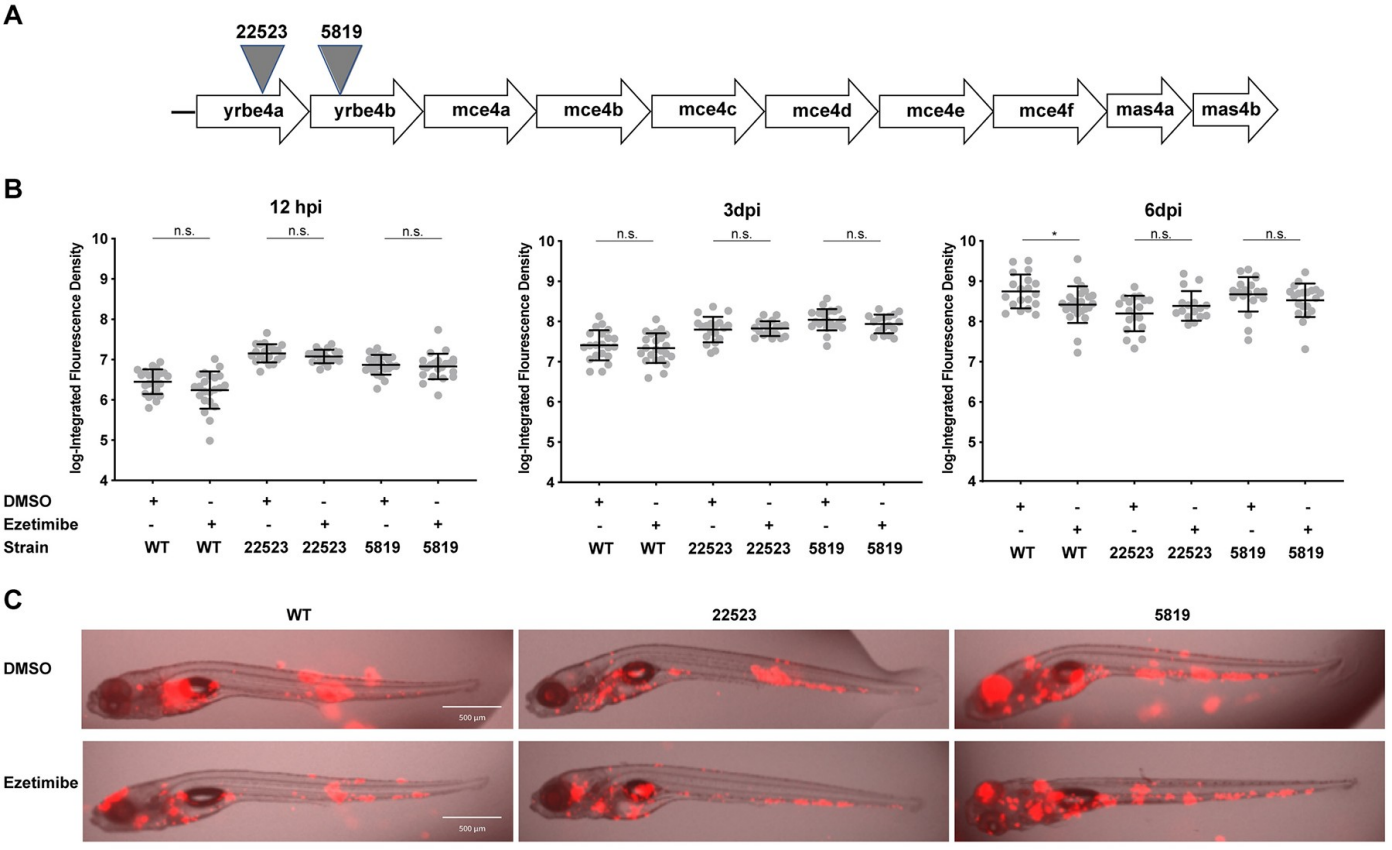
Mutations at the *mce4* locus eliminate the effect of ezetimibe on infection burden. **a**. Schematic representation of *mce4* locus showing location of transposon insertion mutants. **b**. Comparison of infection burden at 12hpi, 3dpi, and 6dpi in animals infected with wild-type, *yrbe4a*^*Tn22523*^, or *yrbe4b*^*Tn5819*^ and then treated with vehicle alone (DMSO) or 1 μM ezetimibe. Student’s t-test *p = 0.021. Representative of three independent experiments. **c**. Median animals from 6dpi comparison groups in (b). Bar = 500μm.

We used a defined *M*. *marinum* transposon mutant library (kind gift of L. Ramakrishnan and C. Cosma) to isolate transposon mutants in the *mce4* gene locus, concentrating on mutants in the upstream genes of the operon *yrbe4a* and *yrbe4b* [37]. These are conserved integral membrane proteins with homology to the ATP-binding Cassette (ABC) transporters [38]. We looked for mutations in the early part of the operon that would individually disrupt function of the system and be predicted to disrupt the operon, including these transporter proteins and downstream genes within the operon. The mutants, designated as *yrbe4a*^*Tn22523*^ and *yrbe4b*^*Tn5819*^ possess independent transposon insertions ~ 95% and ~12% through the open reading frame for each of these early operon genes respectively (Fig 4A). We constructed fluorescent versions of each transposon mutant strain in order to assess the effect of pharmacological manipulation of available neutral lipids via ezetimibe treatment on infection burden in these strains. While ezetimibe treatment of wildtype *M*. *marinum*-infected zebrafish larvae demonstrated a significant reduction in infection burden by 6 dpi (Figs 3F and 4B), no difference in infection burden was observed in the transposon mutant strains under ezetimibe treatment at 3 or 6 dpi when infections were performed to match burden on day 6 (Fig 4B and 4C) or with burden matching at the time of initial infection (S5 Fig). Notably, *mce4* mutants demonstrated a growth defect when compared to the wildtype strain. A reduction in the fold-change of growth was observed in all mutant infections when compared to wild-type growth from initial infection to 6 dpi.

## Discussion

From their first introduction into the host organism, pathogenic mycobacteria must evade host immune responses while also surviving within the nutritionally constrained environment of the macrophage and later the granuloma. The invading microbes must extract essential nutrients from the host, and extensive literature has shown that host-derived lipids are an important carbon and energy source [11, 39]. Host lipids have been shown to accumulate in both the cell wall and as intracellular inclusions within the infecting bacteria [40, 41]. Cholesterol has been demonstrated to accumulate in mycobacterial cell walls [42] and also shown in numerous studies to be required for optimal growth and persistence of mycobacteria within host animal models (10) as well as for maintenance of virulence [43]. Triglycerides have also been seen to accumulate within mycobacteria and are implicated in latent disease [44]. Taken as a starting point, the importance of lipids as a nutrition source for infecting bacteria encouraged investigation of the mechanisms leading to host lipid availability for infecting mycobacteria at early timepoints and the development of lipid rich environments within the mature granuloma.

The development of foamy, lipid-loaded macrophages within the mycobacterium-induced granuloma is well documented, but the mechanism and timing of development are not fully understood. The mechanism of foam cell formation in atherosclerosis has been described largely *in vitro* and has focused on Toll-Like Receptor mediated upregulation of scavenger receptor expression in macrophages [45]. A similar mechanism involving nuclear receptor mediated upregulation of CD36 expression has been proposed in tuberculosis [46–48], but there are also the homeostatic mechanisms which function to balance lipid influx and efflux by macrophages in *in vivo* systems. Some research has suggested that foam cell formation during mycobacteria infection represents a novel disease-specific process due to the accumulation of triglycerides [49]. Other work argues that foam cell formation results from the accumulation of cholesterol and cholesterol derivatives exclusively or predominantly [50, 51]. The difficulty of differentiating neutral lipids via commonly employed staining techniques and the demonstrated accumulation of triglycerides in addition to cholesterol in atherosclerotic macrophages [52] may indicate that diverse lipid accumulation phenotypes and therefore diverse lipid droplet (LD) types exist during mycobacterial infection.

There is some disagreement as to the functional significance of lipid accumulation and lipid droplet (LD) formation within macrophages during mycobacterial infection. Two alternate theories suggest that (1) lipid accumulation is a pathogen directed process with the aim of sequestering host lipids for nutritional use [12, 48] or (2) LDs form as a result of immune activation of host macrophages and as a host-directed defense mechanism [53]. However, as LDs can function as both critical intracellular lipid storage organelles, and in important host immune functions [54] this division of theories may be unnecessary. Central to an understanding of the roles of lipid droplets and accumulated lipids within macrophages during mycobacterial infection is an improved understanding of the natural history and progression of these processes in *in vivo* systems and in real time. To this end, we used the unique capacity of the zebrafish model for intravital imaging and cell-specific sorting to develop novel techniques which allowed us to examine lipid accumulation on the basis of individual macrophage infection status, and to track the very early stages of lipid accumulation in a developing, live infection.

We found that lipid accumulation occurs cell-autonomously in infected macrophages compared to uninfected macrophages within a single infected animal. This observation might indicate that, at early timepoints, infection induces specific changes only in infected macrophages and that the observed accumulation of lipids is not a consequence of a systemic immune activation but rather a pathogen-directed process. Though these results might appear to be in contradiction to findings that lipid droplets form within macrophages following infection as part of a host defense mechanism driven by IFN-gamma and HIF-1alpha dependent signaling [53], it is possible that diverse mechanisms might result in diverse lipid accumulations or lipid droplets within host cells and that some lipid accumulations might be used as nutrition sources and others as immune-active scaffolds. This would reconcile prior findings that mycobacteria are able to acquire host lipids (which would necessarily be stored as intracellular accumulations) but do not acquire these lipids in the presence of IFN-gamma induced LDs [53]. The zebrafish larval system, while providing high *in vivo* resolution of mycobacterial infection, includes only innate immune responses; as in mammals, adaptive immunity develops later. It will be interesting to examine these responses in adult animals.

Work has shown that mycobacteria require cholesterol and lipid utilization genes such as those of the *mce4* operon for optimal growth and persistence *in vivo* [9, 36, 55]. These studies find that the requirement for cholesterol and lipid utilization is observed at timepoints consistent with the onset of adaptive immunity and IFN-γ production. In our studies, lipid accumulation within macrophages began to occur within the first 48 hours of infection and before the development of organized granuloma structures within the larvae; bacterial growth effects using the drug ezetimibe could be observed at 6 days post-infection. These findings suggest that alterations in macrophage lipid content and/or distribution can occur shortly after macrophages become infected with mycobacteria and may also contribute to lipid accumulation at later timepoints. However, necrosis itself within mature granulomas has also been reported to induce metabolic changes in neighboring macrophages to further alter lipid composition [56]. The induction of lipid accumulation within macrophages, whether a pathogen- or host immune-directed process, serves as a plausible additional mechanism by which lipids are enriched in the granuloma and contribute to a nutrient rich environment for mycobacterial growth.

Our data show that lipids accumulating within infected macrophages from early timepoints in infection can be broadly classified as neutral lipids, a group of lipids which includes both TAGs and cholesterol esters. Although we did not directly observe the accumulation of free cholesterol as measured by filipin staining, the storage of intracellular cholesterol deposits in the form of cholesterol esters rather than as free cholesterol is consistent with the ways in which cholesterol is typically stored outside of membrane structures within cells [57] and therefore may represent the species likely to accumulate and be available to intracellular mycobacteria during early infection. TAGs are also known to accumulate within lipid droplets intracellularly frequently in the same cells accumulating CE-rich LDs [58]. Classically, different lipid species can be identified via the use of specific fluorescent probes but the faithful differentiation of neutral lipid species is difficult via these mechanisms [28]. Thus, while the zebrafish model can be used to quantitate and observe lipid accumulation in vivo, it remains for further studies to definitively identify the precise species of the neutral lipids that are present and play functional roles.

Clinical trials of statins, widely-used drugs that inhibit the HMG-CoA reductase required for an upstream step in cholesterol biosynthesis, have been initiated on the basis of research showing that statin therapy reduced bacterial load within macrophages *in vitro* and in mice and that statins may function as adjuvant therapies for tuberculosis [59–61]. However, in pre-clinical models, statins’ therapeutic effects appear to result largely from a discrete mechanism of enhanced autophagy and phagolysosome fusion [62].

Here, we used ezetimibe treatment as an alternate approach, reducing endogenously available neutral lipids during early infection in order to observe the effect on lipid accumulation within macrophages and growth of infecting mycobacteria. We demonstrated that treatment of infected zebrafish with ezetimibe reduced intracellular neutral lipid accumulation and led to a corollary reduction in bacterial burden as compared to untreated zebrafish, suggesting that lipid accumulation does provide a growth advantage for infecting mycobacteria. In contrast to wildtype *M*. *marinum*, mutants deficient in genes of the *mce4* operon were not affected by treatment with ezetimibe. These results suggest that mycobacteria are able to use alternate energy sources during early infection, but that the ability to utilize available lipids can confer a growth advantage even in early infection.

Taken together these data provide evidence that strategies to treat mycobacterial infections through reduction of host lipid availability may be productive. A more nuanced understanding of lipid accumulation, particularly in early infection, may also suggest roles in both pathogen nutrition and host immune defense. The further investigation of the mechanism of lipid accumulation throughout the course of mycobacterial infection may provide targets for drug development and the introduction of adjuvant therapies to supplement current tuberculosis therapies through modulation of the availability of host lipids to invading mycobacteria.

## Methods

### Zebrafish husbandry and handling

All zebrafish husbandry and experimental procedures were performed in accordance with policies approved by the Duke University Institutional Animal Care and Use Committee (Protocol A122-17-05).

Adult zebrafish used to produce larvae for use in these experiments were housed with tankmates of the same strain type or transgenic genotype in 3 or 10L tanks with continuous water flow. Water conditions were maintained at 28°C, conductivity 600–700μS (maintained via Instant Ocean Sea Salt), and pH 7.0–7.3. Fish were fed twice daily with a mix of lab grown Artemia and GEMMA micro 300 (Skettering, Westbrook, Maine).

Larval zebrafish were obtained in clutches via natural spawning and raised in sterile E3 media (5mM NaCl, 178μM KCl, 328 μM CaCl_2_, 400 μM MgCl_2_) at a temperature of 28.5°C with a maximum density of 50 larvae per 50ml petri dish. Larvae were transferred to E3 media containing 50ug/ml 1-phenyl-2-thiourea (PTU, Sigma Aldrich) at 1 dpi in order to arrest pigment development.

Larval zebrafish were anesthetized for all manipulations such as microinjection, imaging, or transfer with tricaine (MS-222; Sigma Aldrich; final concentration 160 ug/ml). Animals were not left in tricaine for longer than 30 minutes if they were to be recovered. All larvae were euthanized on or before 8 dpf by anesthesia followed by cold exposure.

### Mycobacterium marinum

All strains used were derived from the *M*. *marinum* M strain. Bacteria were cultured on either 7H10 agar supplemented with Middlebrook OADC growth supplement (10% v/v; Sigma Aldrich) and 50 μg/ml Hygromycin B or liquid 7H9 media supplemented with Middlebrook OADC growth supplement (10% v/v), 0.05% Tween 80 (Sigma Aldrich), and 50 μg/ml Hygromycin B (7H9 Complete). These media were supplemented with appropriate antibiotics for selection when necessary.

Wildtype *M*. *marinum* strains used in this study were previously described by our group [21]. Wildtype strains were engineered to express TdTomato, Wasabi, or Cerulean fluorescent proteins and Hygromycin B resistance genes. Transposon mutant *M*. *marinum* with disruptions in the yrbe4a and yrbe4b genes of the Mce4 operon (*yrbe4a*^*Tn22523*^ and *yrbe4b*^*Tn5819*^) were identified from a sequenced library of *M*. *marinum* transposon mutants (C. Cosma and L. Ramakrishnan) [37]. The previously described insertion sites were confirmed by PCR and sequencing. Forward and reverse primers were designed for use in pairs with the TnMarR3 primer that anneals within the transposon and directs amplification across the 3’ junction with genomic DNA. For *yrbe4a*^*Tn2252*^, the F primer was 5’- ATCCAACAACTTGCGGTTCC- 3’ and R primer was 5’—GACATGAACCGGTGAAAGCG—3’. For *yrbe4b*^*Tn5819*^, the F primer was 5’- GAGAATCTCGGCCCGGTAAC- 3’ and R primer was 5’—CTGCCATAGCCCTATCGAGTC—3’. Sequencing of amplicons using TnMarR3 confirmed disruptive insertions into the ORF for both strains.

Fluorescent transposon mutant strains were generated via electroporation at 800 Ω, 25 μF, 2.5 kV, 0.2 cm gap with plasmid *msp12*:*mTdTomato-KanR*. Resultant bacteria were plated on 7H10 plates described above and containing 20 μg/ml kanamycin. Colonies were selected and grown in 7H9 Complete media supplemented with kanamycin to confirm TdTomato fluorescence and kanamycin resistance.

*M*. *marinum* samples were prepared for use in infections as previously described. Briefly, bacteria were cultured in 7H9 Complete medium with appropriate antibiotics at 33 C to an OD_600_ of 0.7–1. Cells were pelleted from culture by centrifugation at 4000xg for 15 minutes and then resuspended in 10ml PBST (150 mM NaCl, 8 mM Na2PO4, 2 mM KH2PO4, 3 mM KCl and 0.05% Tween-20; pH 7.4) followed by homogenization via a 27G needle. Cells were again pelleted via centrifugation and then resuspended in 2ml of freezing 7H9 (7H9 supplemented with 10% v/v Middlebrook OADC). The resuspended cells were then divided into five 400μl aliquots and homogenized again via a 27G needle ten times. Each aliquot was then diluted with 1 ml of freezing 7H9 and centrifuged at 770x g for 1 minute resulting in a pellet containing clumped bacteria and a cloudy supernatant of single-cell bacteria. The cloudy supernatants (1 ml each) were collected and placed in a clean tube. The sample was again centrifuged at 770x g for 1 minute, cloudy supernatants were collected and pooled and then filtered through a 5 μm filter. Filtered supernatant was then placed into 1.5 ml conical tubes and cells were pelleted at 13500 rpm for 5 minutes. Pellets were resuspended in 7H9 for freezing, diluted to a cell count of 5 x10^8^ cells per ml, and aliquoted in 5μl quantities for storage at -80°C.

### Infection by microinjection

Frozen *M*. *marinum* aliquots were thawed on ice and diluted with 35ul of 0.5% phenol red solution in PBS (Sigma Aldrich). Larvae were anesthetized at 2dpf and injected with approximately 200 live fluorescent bacteria in a bolus of ~10 nl directed into the caudal vein. Levels of infection were assessed by epifluorescence microscopy and adjusted in order to allow for different infection levels by *M*. *marinum* strain if experimentally required (e.g. Fig 4). For Fig 4, an approximately 2-fold increase in initial burden was established in the initial infections, with 100–200 CFU injected for the control strain and a dose of 250–500 CFU injected for the transposon mutants. Infected embryos were then transferred back to fresh E3 media supplemented with PTU. After 6–12 hours of recovery, larvae were imaged to ensure that no statistically significant infection differences among groups were observed, except for Fig 4, where there were intentionally different starting doses, and these were reflected at this timepoint. Except for intentional differences in initial infection burden, experiments in which unequal initial infections in comparison groups were observed were not analyzed or included.

### Live imaging

Fluorescent microscopy was performed on a Zeiss Observer Z1 inverted microscope. Live embryos and larvae were anesthetized with tricaine and spotted onto glass slides in E3 media for imaging.

Confocal microscopy was performed on an Andor XD Revolution Spinning Disk upright microscope. Live embryos were anesthetized in 120 μg/ml tricaine and then mounted in 0.75% low melting point agarose on 35mm glass bottom microwell dishes with a 20mm microwell and No. 1.5 coverglass (MatTek Ashland, MA) which was then flooded with E3 media supplemented with PTU and 120 μg/ml tricaine.

Fluorescent images were processed and analyzed using Zen 2.5 imaging software (Zeiss). In order to quantify infection burden images were taken using a 2.5x objective and the area and mean intensity of fluorescence were measured and used to calculate the log-Integrated Density of fluorescence which has previously been shown to correlate to infection burden [63]. Quantification of either Nile red or filipin staining in whole larvae was performed by imaging animals using a 2.5x objective and then measuring log-ID of fluorescence for either Nile red (TdTomato) or Filipin (DAPI) in the region of the animal from the anus to the end of the tail.

### Larval dissociation for fluorescence activated cell sorting

Roughly 1000 *mfap4*:*tdTomato* expressing larval zebrafish were infected with Mm:Turq2 and grown in petri dishes with E3 media supplemented with PTU. At 48 hours post infection (hpi) larvae were pooled and euthanized with tricaine and then incubated in deyolking buffer (55 mM NaCl, 1.8mM KCl and 1.25 mM NaHCO_3_) for 5 minutes while hand mixing. Larvae were then separated into 15 ml conical tubes in groups of 100 animals and incubated on ice. Deyolking buffer was then removed and fish were resuspended in FACS buffer without EDTA [5% heat-inactivated fetal bovine serum and 10 mM HEPES in HBSS (Gibco)]. Larvae were then dissociated by adding Liberase (Roche, 2μg/ml final concentration), Collagenase XI (Sigma, 6 U/ml final), Hyaluronidase 1a (Sigma 3 U/ml final) and DNaseI (Sigma 1μg/ml) followed by trituration for 1 minute via pipetting with a 1000μl tip and incubation for 5 minutes at 30 °C on an orbital platform at 75 RPM. The mechanical disruption steps were repeated 6 times at which point additional enzyme was added and dissociation steps were repeated again for an additional 6–10 times until all visible chunks were eliminated. To stop enzymatic digestion, 120mM EDTA (in HBSS with 10mM HEPES) was added to a final concentration of 5mM EDTA and dissociated pools were recombined. FACS buffer with EDTA [5% heat-inactivated fetal bovine serum, 10 mM HEPES, and 2mM EDTA in HBSS (Gibco)] was added to the dissociated cells to a volume of 20ml. The cell solution was then filtered through a 30 μm cell strainer (Miltenyi Biotec) into 50 mL conical tubes and the filter was then washed with an additional 10 mL of FACS buffer with EDTA. Cells were pelleted by centrifugation for 10 minutes at 2000 rpm. The supernatant was decanted and cells were the resuspended in 0.5–1 mL of FACS buffer with EDTA depending on pellet size and then transferred to FACS tubes. DNaseI (5μg/mL) and 7-AAD (5μg/mL) were added to samples. Sorting of non-fluorescent cells, fluorescent red macrophages and fluorescent red macrophages infected by cerulean *M*. *marinum* was performed on the Beckman Coulter Astrios cell sorter at the Duke Cancer Institute Flow Cytometry Shared Resource. Single color control samples were used to set up compensation and gating.

### Lipid staining and quantification

Sorted cell types from larval infections were pelleted by centrifugation for 10 minutes at 2000 rpm and then resuspended in PBS with 1% paraformaldehyde for fixation. To quantify intracellular neutral lipid 10 mg/ml Nile red (Sigma) in acetone (0.5 μg/mL final concentration) was added to fixation solution immediately and then incubated for 30 minutes while protected from light. To quantify intracellular free cholesterol fixation was allowed to proceed for 30 minutes before 25 mg/ml Filipin III (Sigma) in DMSO (0.05 mg/ml final concentration) was added to the fixation solution and incubated for 1 hour protected from light. Following staining, cell populations were washed 3 times with PBS and then analyzed on the BD Biosciences Fortessa X-20 analyzer at the Duke Cancer Institute Flow Cytometry Shared Resource. Nile red stained samples were analyzed via excitation at 488 nm with collection by a 586/15 bandpass filter. Filipin stained samples were analyzed via excitation at 360nm with collection by a 515/30 bandpass filter. Data were analyzed with FloJo v10 (Treestar, CA). For each data set corrected fluorescence intensity was computed by subtracting the geometric mean fluorescence intensity of non-fluorescent cells from the geometric mean fluorescence intensity of the test cells (uninfected or infected macrophages). These corrected intensities were then used to calculate fold change of fluorescence between uninfected and infected macrophages stained with Nile red or Filipin.

### Tail fin infection, staining, and imaging

Frozen *M*. *marinum* aliquots were thawed on ice and diluted with 25ul of 0.5% phenol red solution in PBS (Sigma Aldrich). Larvae were anesthetized at 3 dpf and infected by inserting a borosilicate glass microcapillary pulled into a needle into the tail fin (29) at the body junction and slowly injection until a region of the fin can been seen to be filled with dye (Fig 2) indicating that *M*. *marinum* particles have been deposited between the 2 epidermal layers of the fin. Larvae were then washed and returned to fresh E3 media supplemented with PTU. Larvae were allowed to recover and grow for 48 hrs at which time larvae were imaged by epifluorescence in order to confirm infection.

Infected larvae at 2 dpi were transferred to E3 media supplemented with PTU and 0.5 ug/mL Nile red dye. Larvae were allowed to swim in the supplemented media for 30 minutes protected from light. Larvae were then washed 3x by removing from the dye containing media and placing the larvae in fresh E3 supplemented with only PTU, allowing the animals to rest for 5 minutes between each wash. Larvae were them prepared for live confocal imaging as described above. Larvae were imaged at the infection sight by both 20x water immersion and 63x silicone oil immersion.

Quantification of *in vivo* neutral lipid accumulation was performed using ImageJ (NIH). For each animal, image stacks were used to classify infected versus uninfected macrophages visually. Image stacks were then flattened by maximum intensity projection in the Nile red channel and each macrophage was tagged as a region of interest (ROI). The Integrated Fluorescent Density (IFD, area x mean fluorescent intensity) was calculated for each ROI and then matched to its macrophage classification. The average IFD for uninfected vs infected macrophages was then determined for each larvae and plotted for comparison. The fold change of IFD was calculated for each larva individually comparing the IFD and log-IFD of infected versus uninfected macrophages.

### Infection and lipid quantification in the tail fin under ezetimibe treatment

In order to assess the effect of ezetimibe treatment on neutral lipid and free cholesterol levels in larvae, fish were grown to 3 and 5 dpf in E3 media supplemented with PTU and either 0.5% DMSO or 1uM ezetimibe in DMSO. Ezetimibe treatment was started at 2 dpf. Larvae were either stained with Nile red as described above or euthanized and fixed with 3.7% paraformaldehyde in PBS by rocking at 4°C overnight. After fixation larvae were removed from fixation solution and washed three times in PBS followed by staining in 0.05 mg/ml filipin while protected from light. Staining was performed at 4°C while rocking overnight. Following staining fixed larvae were washed and imaged by fluorescent microscopy.

The effect of ezetimibe treatment was further assessed by treating animals with either 0.5% DMSO or 1uM Ezetimbe in DMSO beginning at 2 dpf. Treated fish were then infected in the tail fin, stained, and imaged as described above. Differences in neutral lipid accumulation at the foci of infection were compared visually.

### *In vitro* growth analysis

A starter culture of *M*. *marinum* M strain expressing TdTomato was generated by inoculating 1 mL 7H9 complete media with -80C freezer stock and then allowing cultures to grow at 28.5C until reaching stationary phase. The starter culture was pelleted by centrifugation and then resuspended in fresh 7H9 complete medium. The bacteria were then diluted to a starting OD_600_ of approximately 0.035 with either 0.5% DMSO or 1 μM ezetimibe in 0.5% DMSO. OD_600_ readings were taken at 24 hour intervals for the next 96 hours.

### Statistics

Statistical testing was performed using GraphPad Prism 7. The specific test(s) performed are noted in the figure legends for each respective analysis.

## Supporting information

S1 Fig Related to Fig 1. Neutral lipids accumulate cell-autonomously in *M*. *marinum*-infected macrophages as measured by FACS. **a**. Measurement of Nile red fluorescence in sorted cell populations. A 4.0-fold increase was observed in the geometric mean fluorescence intensity of Nile red staining between uninfected (red) and infected (cerulean) macrophages. **B**. Measurement of Nile red fluorescence in sorted cell populations. In an independent replicate, a 3.5-fold increase was observed in the geometric mean fluorescence intensity of Nile red staining between uninfected (red) and infected (cerulean) macrophages.
(TIF)

S2 Fig Related to Fig 2. Neutral lipid accumulation appearance within infected macrophages. **a**. Confocal imaging of a representative *Tg(mfap4*:*p2A-Turquoise2)* larva 2 dpi with *M*. *marinum* (green) and following staining with Nile red showing detail of Nile red staining pattern. 60x objective, bar = 20 μm. **b**. Confocal imaging of a representative *Tg(mfap4*:*p2A-Turquoise2)* larva 2 dpi with *M*. *marinum* (green) and following staining with Nile red showing detail of Nile red staining pattern. 60x objective, bar = 20 μm. **c**. Confocal imaging of a representative *Tg(mfap4*:*p2A-Turquoise2)* larva 2 dpi with *M*. *marinum* (green) and following staining with Nile red showing detail of Nile red staining pattern. 60x objective, scale bar = 20 μm. **d**. Confocal imaging of a representative *Tg(mfap4*:*p2A-Turquoise2)* larva 2dpi with *M*. *marinum* (green) and following staining with Nile Red showing detail of Nile red staining pattern. 60x objective, scale bar = 20 μm.
(TIF)

S3 Fig Related to Fig 2. Neutral lipids do not accumulate in macrophages following mock infection. **a**. Confocal imaging of tail fin of *Tg(mfap4*:*p2A-Turquoise2)* larvae 2 days post mock infection via injection of phenol red and staining with Nile red. Absence of Nile red stain accumulation. 20x objective, scale bar = 100 μm.
(TIF)

S4 Fig Related to Fig 3. Treatment with ezetimibe reduces neutral lipid accumulation at infection foci. **a**. Comparison of integrated mean fluorescence at the focus of infection in animals treated with vehicle alone (0.5% DMSO) or 1 μM ezetimibe in vehicle. The focus of infection was defined as the region of macrophage accumulation at the site of infection. The mean fluorescence of Nile red signal was determined by drawing a region around the macrophage accumulation and then measuring Nile red fluorescence signal within this region and calculating mean fluorescence. Animals treated with ezetimibe showed reduced accumulation of Nile red signal at the focus of infection by comparison of mean fluorescence by Student’s t-test, p = 0.0076.
(TIF)

S5 Fig Related to Fig 4. Burden matching of initial infections demonstrates a growth defect in transposon mutants compared to wildtype growth but loss of the effect of ezetimibe on infection burden. **a**. Comparison of infection burden at 12 hpi, 3 dpi, and 6 dpi in animals infected with wild-type, *yrbe4a*^*Tn22523*^, or *yrbe4b*^*Tn5819*^ and then treated with vehicle alone (DMSO) or 1 μM ezetimibe. Infection burdens at the initial timepoint showed no difference in burden at 12 hpi between wild-type, *yrbe4a*^*Tn22523*^, or *yrbe4b*^*Tn5819*^. By 3 dpi a burden difference was observed between wildtype and *yrbe4a*^*Tn22523*^ (p<0.0001) but not between wildtype and *yrbe4b*^*Tn5819*^. By 6dpi differences were observed between wildtype and both *yrbe4a*^*Tn22523*^ and *yrbe4b*^*Tn5819*^ (p<0.0001) all in untreated strains. Reduced burden is observed between ezetimibe-treated and untreated wildtype samples (p = 0.003).
(TIF)
