# Supplementary figures and images for: Early cell-autonomous accumulation of neutral lipids during infection promotes mycobacterial growth
Source: PLoS One. 2020 May 14;15(5):e0232251. doi: 10.1371/journal.pone.0232251 (PMC7224534; doi:10.1371/journal.pone.0232251)

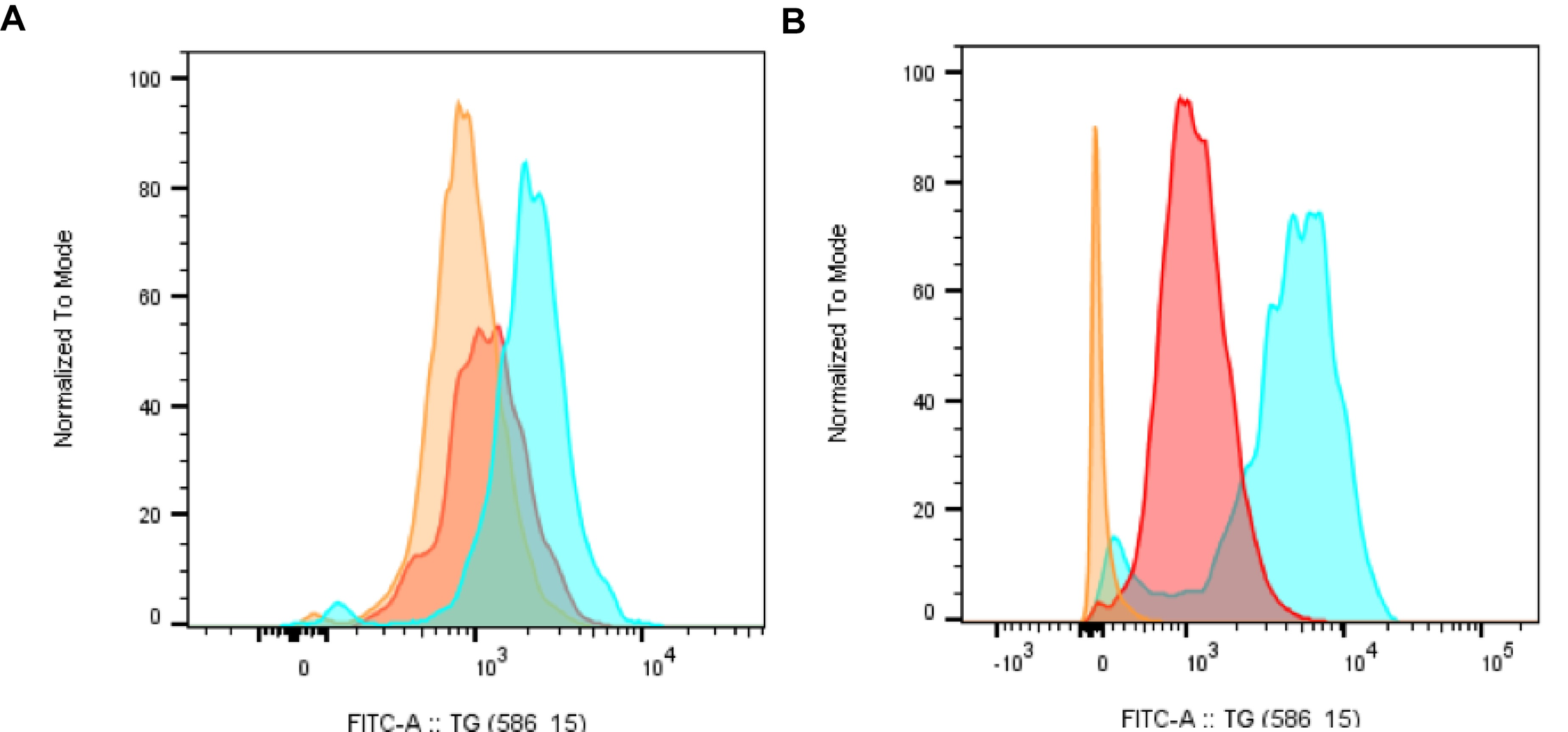

Supplement: S1 Fig — a. Measurement of Nile red fluorescence in sorted cell populations. A 4.0-fold increase was observed in the geometric mean fluorescence intensity of Nile red staining between uninfected (red) and infected (cerulean) macrophages. B. Measurement of Nile red fluorescence in sorted cell populations. In an independent replicate, a 3.5-fold increase was observed in the geometric mean fluorescence intensity of Nile red staining between uninfected (red) and infected (cerulean) macrophages. (TIF) [file pone.0232251.s001.tif]

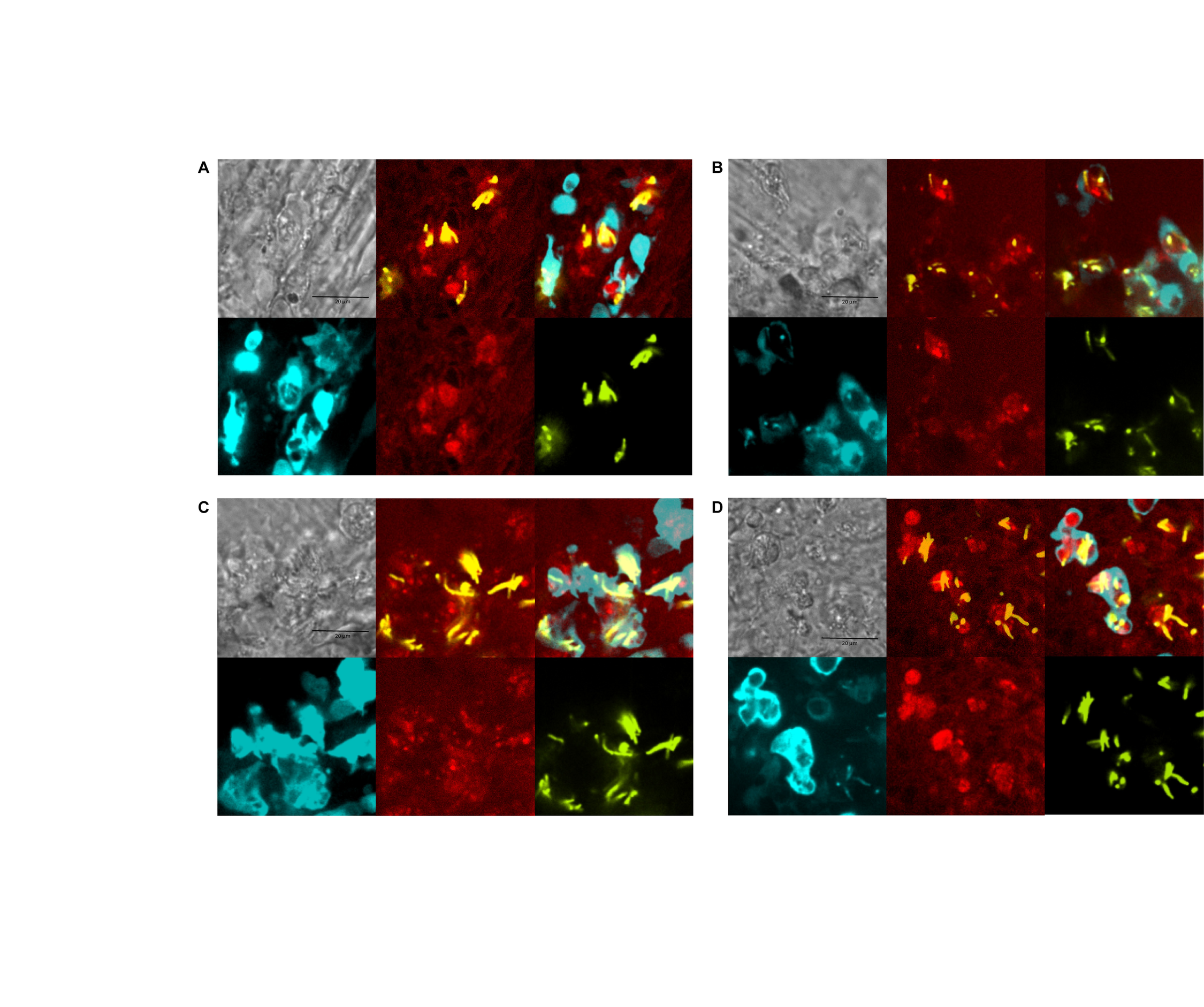

Supplement: S2 Fig — a. Confocal imaging of a representative Tg(mfap4:p2A-Turquoise2) larva 2 dpi with M. marinum (green) and following staining with Nile red showing detail of Nile red staining pattern. 60x objective, bar = 20 μm. b. Confocal imaging of a representative Tg(mfap4:p2A-Turquoise2) larva 2 dpi with M. marinum (green) and following staining with Nile red showing detail of Nile red staining pattern. 60x objective, bar = 20 μm. c. Confocal imaging of a representative Tg(mfap4:p2A-Turquoise2) larva 2 dpi with M. marinum (green) and following staining with Nile red showing detail of Nile red staining pattern. 60x objective, scale bar = 20 μm. d. Confocal imaging of a representative Tg(mfap4:p2A-Turquoise2) larva 2dpi with M. marinum (green) and following staining with Nile Red showing detail of Nile red staining pattern. 60x objective, scale bar = 20 μm. (TIF) [file pone.0232251.s002.tif]

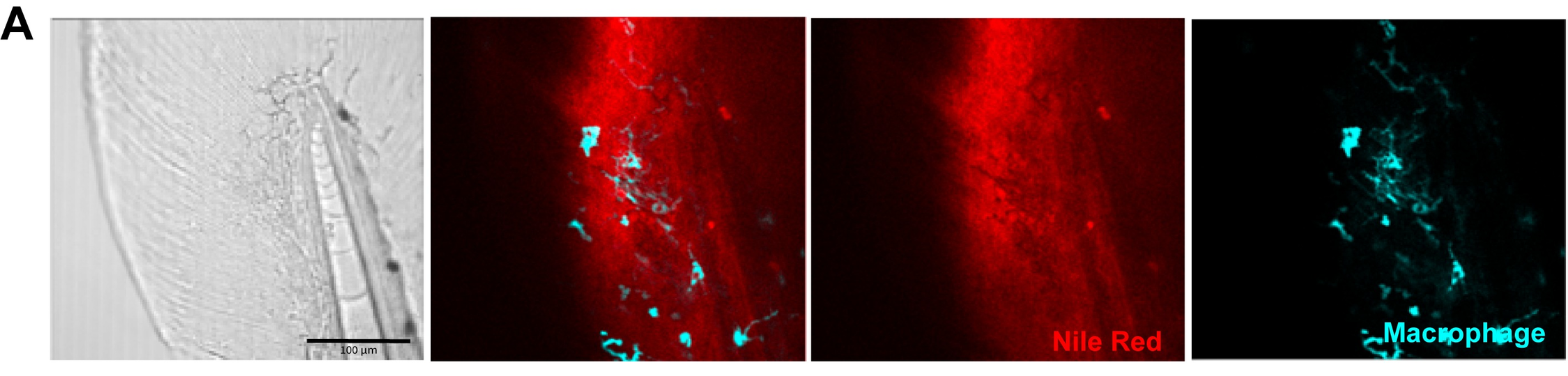

Supplement: S3 Fig — a. Confocal imaging of tail fin of Tg(mfap4:p2A-Turquoise2) larvae 2 days post mock infection via injection of phenol red and staining with Nile red. Absence of Nile red stain accumulation. 20x objective, scale bar = 100 μm. (TIF) [file pone.0232251.s003.tif]

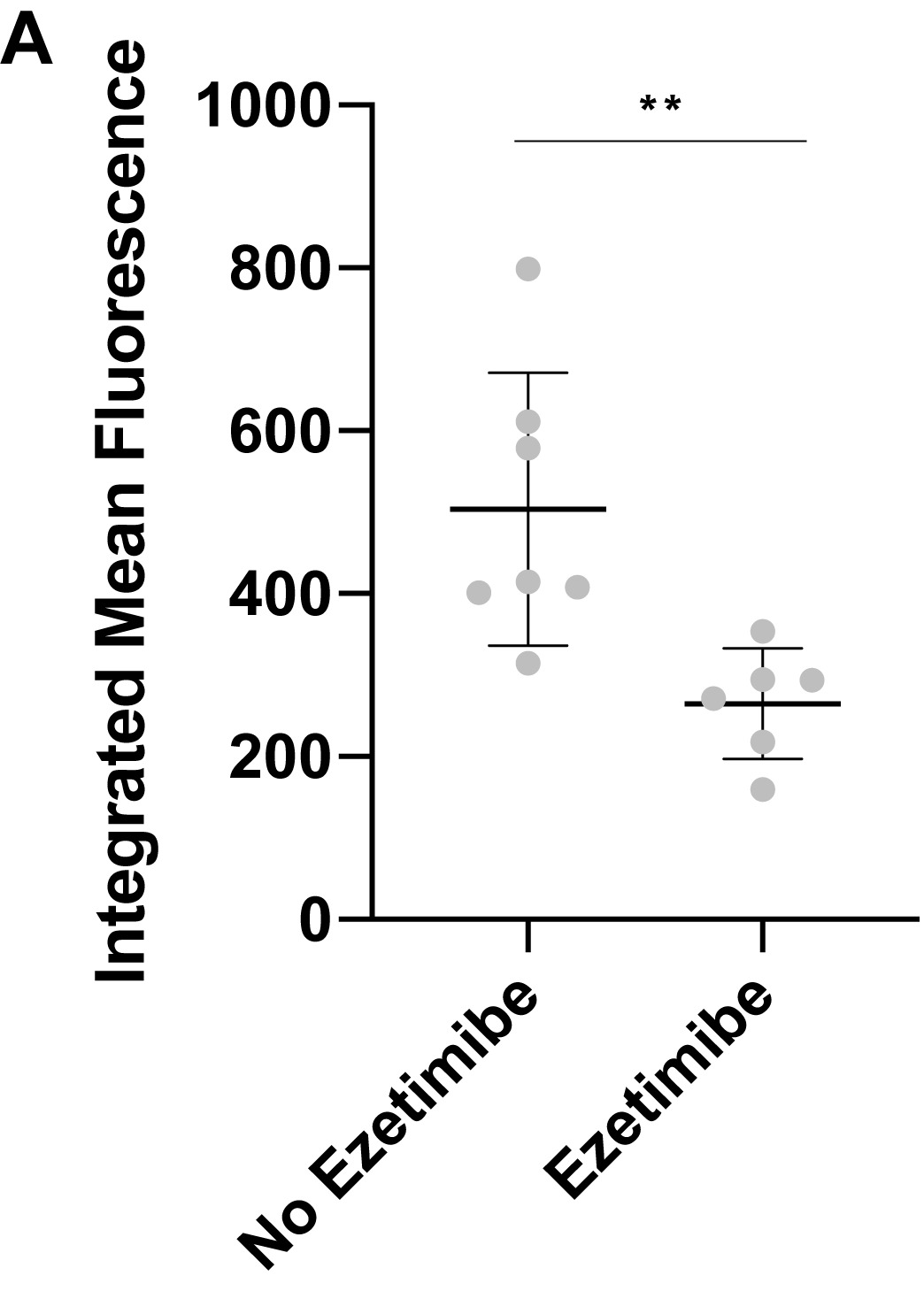

Supplement: S4 Fig — a. Comparison of integrated mean fluorescence at the focus of infection in animals treated with vehicle alone (0.5% DMSO) or 1 μM ezetimibe in vehicle. The focus of infection was defined as the region of macrophage accumulation at the site of infection. The mean fluorescence of Nile red signal was determined by drawing a region around the macrophage accumulation and then measuring Nile red fluorescence signal within this region and calculating mean fluorescence. Animals treated with ezetimibe showed reduced accumulation of Nile red signal at the focus of infection by comparison of mean fluorescence by Student’s t-test, p = 0.0076. (TIF) [file pone.0232251.s004.tif]

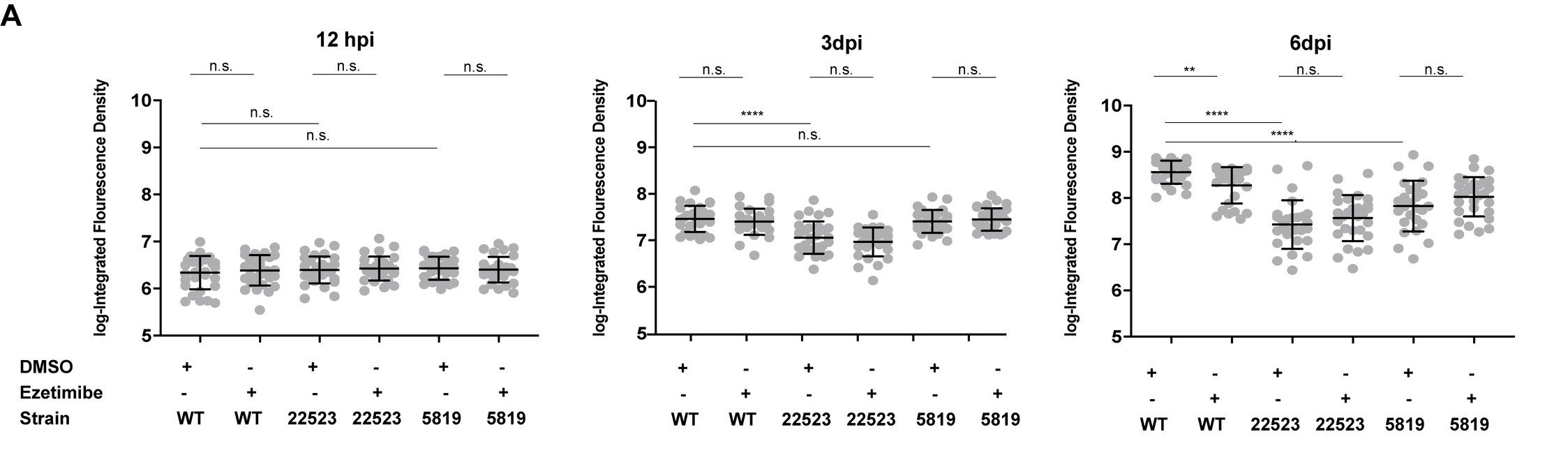

Supplement: S5 Fig — a. Comparison of infection burden at 12 hpi, 3 dpi, and 6 dpi in animals infected with wild-type, yrbe4aTn22523, or yrbe4bTn5819 and then treated with vehicle alone (DMSO) or 1 μM ezetimibe. Infection burdens at the initial timepoint showed no difference in burden at 12 hpi between wild-type, yrbe4aTn22523, or yrbe4bTn5819. By 3 dpi a burden difference was observed between wildtype and yrbe4aTn22523 (p<0.0001) but not between wildtype and yrbe4bTn5819. By 6dpi differences were observed between wildtype and both yrbe4aTn22523 and yrbe4bTn5819 (p<0.0001) all in untreated strains. Reduced burden is observed between ezetimibe-treated and untreated wildtype samples (p = 0.003). (TIF) [file pone.0232251.s005.tif]
